# Upon the Inefficacy of Phosphate of Lime

**Published:** 1855-10

**Authors:** 


*Upon the inefficacy of Phosphate of Lime.*

Experiments have been instituted in the Bethany Hospital, at Berlin, with reference to the use of phosphate of lime recommended by Beneke, in cases of atrophical children, persons with scrofulous affections, caries of the joints, suppurating lymphatic glands, spina ventosa, &c. Not the slightest improvement, not even the amelioration of a symptom, was evidenced. In every instance, an increase of the affection was observed. The preparation ordered was from two to four grains three times daily, which was continued without interruption for eight weeks. —*Günns. Zetschr.* v. 6. 1854., *Schmidt's Jahr.*

